# Single-cell RNA sequencing of circulating immune cells supports inhibition of *TNFAIP3* and *NFKBIA* translation as psoriatic arthritis biomarkers

**DOI:** 10.3389/fimmu.2025.1483393

**Published:** 2025-02-07

**Authors:** Ameth N. Garrido, Rohan Machhar, Omar F. Cruz-Correa, Darshini Ganatra, Sarah Q. Crome, Joan Wither, Igor Jurisica, Dafna D. Gladman

**Affiliations:** ^1^ Gladman-Krembil PsA Research Program, Schroeder Arthritis Institute, Krembil Research Institute, University Health Network, Toronto, ON, Canada; ^2^ Ajmera Transplant Centre, Toronto General Hospital Research Institute, University Health Network, Toronto, ON, Canada; ^3^ Department of Immunology, Faculty of Medicine, University of Toronto, Toronto, ON, Canada; ^4^ Schroeder Arthritis Institute, Krembil Research Institute, University Health Network, Toronto, ON, Canada; ^5^ Division of Rheumatology, Faculty of Medicine, University of Toronto, Toronto, ON, Canada; ^6^ Osteoarthritis Research Program, Division of Orthopedic Surgery, Schroeder Arthritis Institute and Data Science Discovery Centre for Chronic Diseases, Krembil Research Institute, University Health Network, Toronto, ON, Canada; ^7^ Departments of Medical Biophysics and Computer Science, and Faculty of Dentistry, University of Toronto, Toronto, ON, Canada; ^8^ Institute of Neuroimmunology, Slovak Academy of Sciences, Bratislava, Slovakia

**Keywords:** psoriatic arthritis, psoriasis, scRNA-seq, *TNFAIP3*, *NFKBIA*

## Abstract

**Objective:**

To identify biomarkers that distinguish psoriatic arthritis (PsA) from cutaneous psoriasis without arthritis (PsC) and healthy controls (HC) using single cell RNA sequencing (scRNA-seq).

**Method:**

Peripheral blood mononuclear cell samples from three patients with PsA fulfilling CASPAR criteria, three patients with PsC and two HC were profiled using scRNA-seq. Differentially expressed genes (DEGs) identified through scRNA-seq were validated on classical monocytes, and CD4^+^ and CD8^+^ T cell subsets derived from an independent cohort of patients using the NanoString nCounter^®^ platform. Protein expression was measured in CD4^+^ and CD8^+^ T cells by immunoblotting.

**Results:**

A total of 18 immune cell population clusters were identified. Across 18 cell clusters, we identified 234 DEGs. *NFKBIA* and *TNFAIP3* were overexpressed in PsA vs HC and PsC patients. Immunoblotting of the proteins encoded in these genes (IκBα and A20, respectively) showed higher levels in PsA CD4^+^ T cells compared to HC. Conversely, lower levels were observed in PsA CD8^+^ T cell lysates compared to HC for both proteins.

**Conclusion:**

These results suggest that translation of *TNFAIP3* and *NFKBIA* may be inhibited in PsA CD8^+^ T cells. This study provides insight into the cellular heterogeneity of PsA, showing that non-cell type specific expression of genes associated with the disease can be dysregulated through different mechanisms in distinct cell types.

## Introduction

Psoriatic Arthritis (PsA) is an immune mediated inflammatory musculoskeletal disease that adversely impacts quality of life (1–3). It is characterized by asymmetric joint involvement and concomitant psoriasis (4). Comorbidities associated with PsA include depression, cardiovascular disease, and diabetes (5–7). The etiology of PsA remains unknown (8). Risk factors for PsA include a clinical diagnosis of psoriasis, family members with psoriatic disease, obesity, smoking, and HLA-B27 positivity (9, 10). A diagnosis of PsA is heavily reliant on clinical expertise due to the heterogeneous nature of the disease (11). While numerous studies investigated diagnostic, disease activity and treatment response biomarkers for PsA, none have been validated for point of care use (12).Single-cell RNA sequencing (scRNA-seq) studies have found clonal expansions of CD8^+^ T Cells in synovial tissue of PsA patients compared to peripheral blood (13), display an enriched pro-inflammatory Th17 cell transcriptomic signature (14), and enrichment of Tregs and dendritic cells (15).

To get better mechanistic insights into PsA, we aimed to investigate the transcriptional landscape of PsA patients in peripheral blood mononuclear cells (PBMC) to identify potential susceptibility factors for disease. Here we combined scRNA-seq with T cell Receptor (TCR) immune profiling to capture potential clonal expansions of PsA and compare their immune landscape directly to psoriasis and healthy controls. Herein we also provide for the first-time evidence of translation inhibition of two important transcripts associated with inflammation *NFKBIA* and *TNFAIP3* in PsA CD8^+^ T Cells.

## Patients and methods

### Patient selection

All study participants were recruited from the Psoriatic Disease Program at the Toronto Western Hospital, Toronto, Canada. PsA patients satisfied the ClASsification for Psoriatic Arthritis (CASPAR) criteria, had at least one actively inflamed joint and were not on any biologic treatment. Psoriasis without arthritis (PsC) patients had psoriasis diagnosis confirmed by a dermatologist and absence of arthritis by a rheumatologist. Healthy participants declared an absence of any disease or family history of psoriatic disease.

Three PsA patients and three PsC patients matched for age, sex, and psoriasis duration, and two healthy controls (HC) matched by age and sex, were selected for the discovery phase, while an independent cohort of 14 PsA patients, 17 PsC patients and 14 HC were selected for the validation phase (**Table 1**). All PsA patients fulfilled the CASPAR criteria and PsC patients were confirmed by a dermatologist to have psoriasis and by a rheumatologist not to have PsA. PASI was not controlled for, as this was an exploratory analysis entered on differences between PsA and PsC and not between cutaneous psoriasis disease severity. Race and ethnicity were self-reported; all patients were white. None of the patients had any history of other autoimmune diseases. The study was approved by the University Health Network Research Ethics Board (#18-5357) and all participants provided written informed consent.

**Table 1:** Demographics and clinical information of the study groups selected for the discovery phase (scRNA-seq and TCR immune profiling) and the validation phase (gene expression in classical monocytes, CD4^+^ and CD8^+^ T cell subsets and immunoblotting).

	Discovery Phase			Validation Phase		
Variable	PsA (n=3)	PsC (n=3)	HC (n=2)	PsA (n=14)	PsC (n=17)	HC (n=14)
Age	64.8 ± 11	65.13 ± 9.91	57.11 ± 6.08	55.07 ± 12.34	55.5 ± 16.85	42.12 ± 11.67
Sex (%Female)	1 (33.33%)	1 (33.33%)	1 (50%)	7 (50%)	10 (58.82%)	7 (50%)
BMI (kg/m^2^)	28.47 ± 5.92	29.64 ± 8	NA	28.74 ± 4.47	27.96 ± 5.56	NA
PASI	1.43 ± 1.45	3.83 ± 4.44	NA	1.49 ± 2.73	3.41 ± 3.49	NA
Active joints	3.67 ± 3.51	0 ± 0	NA	8 ± 10.52	0.18 ± 0.73	NA
Enthesitis	0 ± 0	0 ± 0	NA	0.57 ± 1.28	0 ± 0	NA
Age of PsC onset (years)	44.67 ± 11.37	43 ± 12.12	NA	26.14 ± 15.64	33 ± 13.89	NA
PsC Duration (years)	20.14 ± 8.3	22.13 ± 10.51	NA	27.98 ± 19.17	17.4 ± 17.39	NA
NSAIDs (%)	1 (33.33%)	0 (0%)	0 (0%)	6 (42.86%)	6 (35.29%)	0 (0%)
DMARDs (%)	1 (33.33%)	1 (33.33%)	0 (0%)	7 (50%)	4 (23.53%)	0 (0%)
Biologics (%)	0 (0%)	0 (0%)	0 (0%)	4 (28.57%)	1 (5.88%)	0 (0%)

Values presented as mean ± standard deviation or number of patients (percentage). PsA, Psoriatic Arthritis; PsC, Cutaneous psoriasis; HC, Healthy Controls; BMI, Body Mass Index; PASI, Psoriasis Area Severity Index; NSAIDs, Non-Steroidal Anti-Inflammatory drugs; DMARDS, Disease Modifying Antirheumatic Drugs.

### Single-cell RNA-sequencing

10x Genomics 5’ scRNA-seq v2 with TCR immune profiling was performed at the Princess Margaret Genomics Centre (PMGC, Toronto, Canada) on frozen unsorted PBMCs isolated by ficoll-paque™ (GE Healthcare) using a standard protocol. Sample preparation and library construction was carried out following the manufacturer’s recommendations and scRNA-seq was performed on an Illumina HiSeq 2500 instrument at a depth of 50,000 reads per cell for gene expression libraries and 5,000 reads per cell for V(D)J TCR enriched libraries.

### scRNA-seq data processing

The sequencing raw base call files were converted to FASTQ files and aligned to the GRCh38 reference human transcriptome using Cell Ranger v.3.1.0. Barcode-feature matrices were imported into R v.3.6.3 for analysis. Cells containing over 2.5 median absolute deviations (MADs) of mitochondrial content, ± 2.5 MADs library size and unique detected genes per cell were removed using Scater v.1.14.0. Normalization was performed using the scran package v.1.14.0 (16). Seurat v.3 (17) was used for dimensional reduction and clustering. In clusters where it was difficult to discern cell type, PanglaoDB v.27/03/2020 was used to detect cell markers for annotation (18). DEGs were detected across the three groups in a pairwise comparison. Pathway enrichment analysis of significant DEGs in each cell-type was performed using pathDIP v.4.0 (19), restricting the analysis to literature-curated pathways and experimentally detected interactions at a threshold of 0.99. Bonferroni correction was used to control for multiple testing.

### scRNA-seq TCR immune profiling analysis

Cell Ranger v.3.1.0 was used to generate paired alpha and beta chain V(D)J single cell sequences. Secondary analysis was conducted in R v.4.0.1 using the following packages: scRepertoire v.1.4.0 (20), immunarch v.0.6.7, dunn.test v.1.3.5 and Seurat v.4 (21). Antigen specificity of expanded clonotypes was predicted using the VDJdb 2022 database (22).

### Isolation of immune cells

CD8^+^ T cells, CD4^+^ T cells and classical monocytes were isolated from peripheral blood using positive selection with CD8 MicroBeads (Milteny Biotec, 130-045-201) and CD4 MicroBeads (Miltenyi Biotec, 130-045-101) and subsequently using the Classical Monocyte Isolation kit (Miltenyi Biotec, 130-117-337) via negative selection, following the manufacturers’ recommendations.

### Immunoblotting

A total of 25ug of proteins was loaded into each well and SDS-PAGE was carried out. Proteins were transferred onto a PVDF membrane using the wet transfer method. Once complete, SYPRO^®^ Ruby Protein Blot Stain (BIO-RAD) was added to perform downstream total protein normalization. Primary antibody incubation for IκBα (mouse mAb #4814, CST^®^) or A20/TNFAIP3 (rabbit mAb #5630, CST^®^) was performed overnight at 4^0^C for 16 hours. Horse-Radish Peroxidase (HRP) linked IgG mouse or rabbit (CST^®^) secondary antibodies were incubated with the blot for 90 minutes at RT under agitation. Cytvia Amersham™ ECL™ Prime Western Blotting Detection Reagent was used to develop the blot.

Protein normalization and band detection was conducted on ImageLab (BIO-RAD). A multi-channel image was created which consisted of the bands for A20, IκBα and SYPRO^®^ Ruby protein blot stain for total protein normalization. In all blots, HaCaT and THP cell lysates (Abcam) were loaded as positive controls. Band intensities were imported into R version v.4.0.1 and a Wilcoxon rank sum test was used to compare protein expression between the groups, when the difference was significant (p<0.05) follow-up pairwise Dunn tests were performed.

### NanoString nCounter^®^ assay

A custom nCounter^®^ CodeSet probe panel (**Supplementary Table 1**) was designed to target differentially expressed genes (DEG) identified by scRNA-seq in the discovery phase. In addition to positive and negative hybridization controls, three housekeeping genes were included: Beta 2 Microglobulin (*B2M*), Glyceraldehyde 3-phosphate dehydrogenase (*GAPDH*) and Beta Actin (*ACTB*). NanoString^®^ assays were carried out at the PMGC on the nCounter^®^ platform using 100 ng total RNA input isolated using TRIzol^®^. RNA was hybridized with probes, loaded onto an nCounter^®^ cartridge well and immobilized prior to being digitally analyzed. Normalized gene expression levels were used for DEG analysis using the three housekeeping genes. A Wilcoxon rank sum test was used to compare the gene expression of all three groups, if p<0.05, pair wise comparisons were made using a Dunn test.

## Results

### Cell types in scRNA-seq dataset

**Figure 1:**
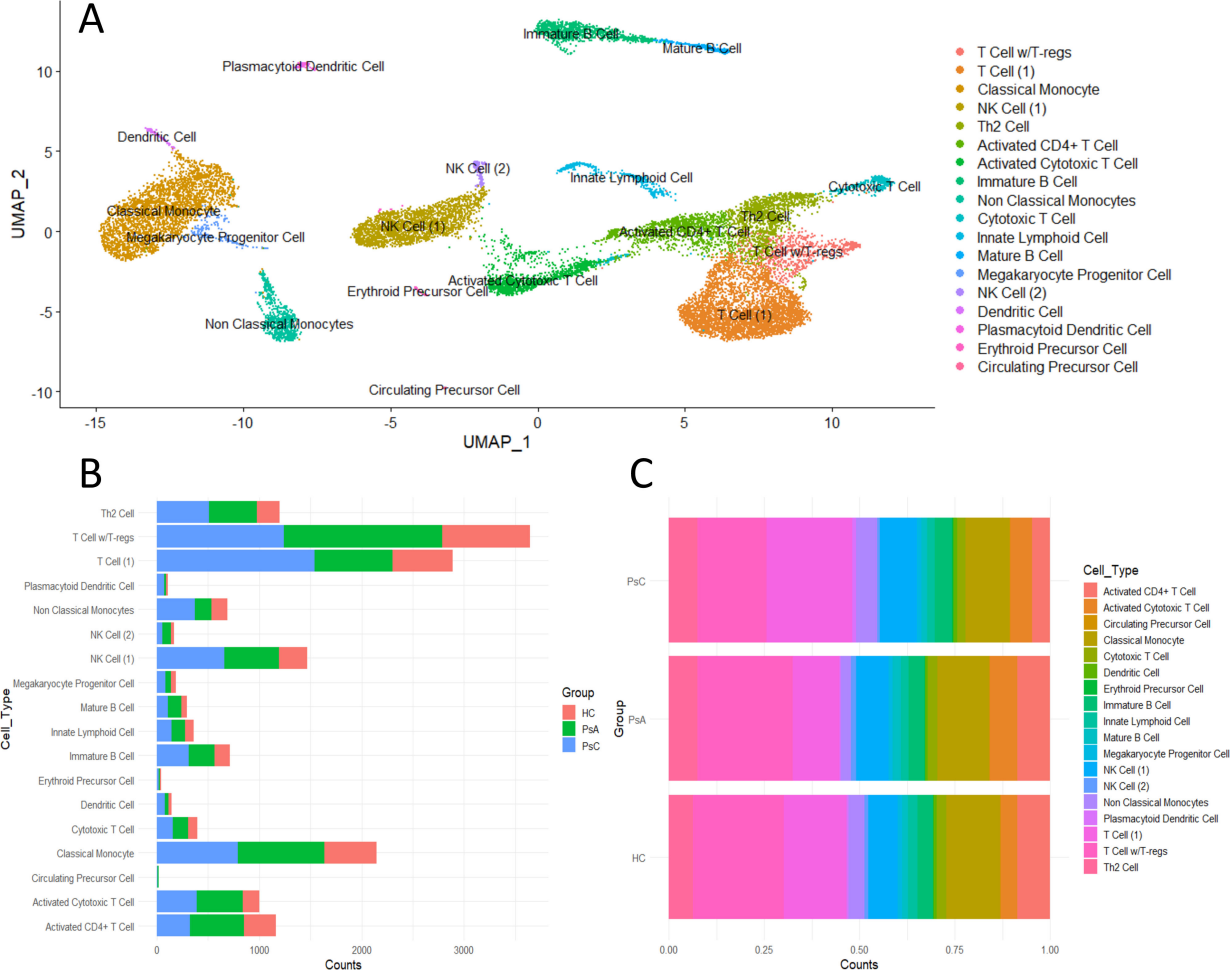
Immune landscape of scRNA-seq PBMC dataset containing 3PsA, 3PsC and 2 HC samples. **(A)** UMAP plot of identified cell clusters. **(B)** Detected cell counts in each cluster per group. **(C)** Proportion of cell clusters by group.

scRNA-seq was performed on three PsC, three PsA and two HC PBMC samples with 18 cell clusters being identified based on unique gene expression profiles (**Supplementary Results**). This included multiple T cell lineages, classical and non-classical monocytes, Natural Killer (NK) cells, B cells, and other populations such as myeloid dendritic cells (**Figure 1**, **Supplementary Figure 1**).

### scRNA-seq differential gene expression analysis

#### Classical Monocyte

**Figure 2:**
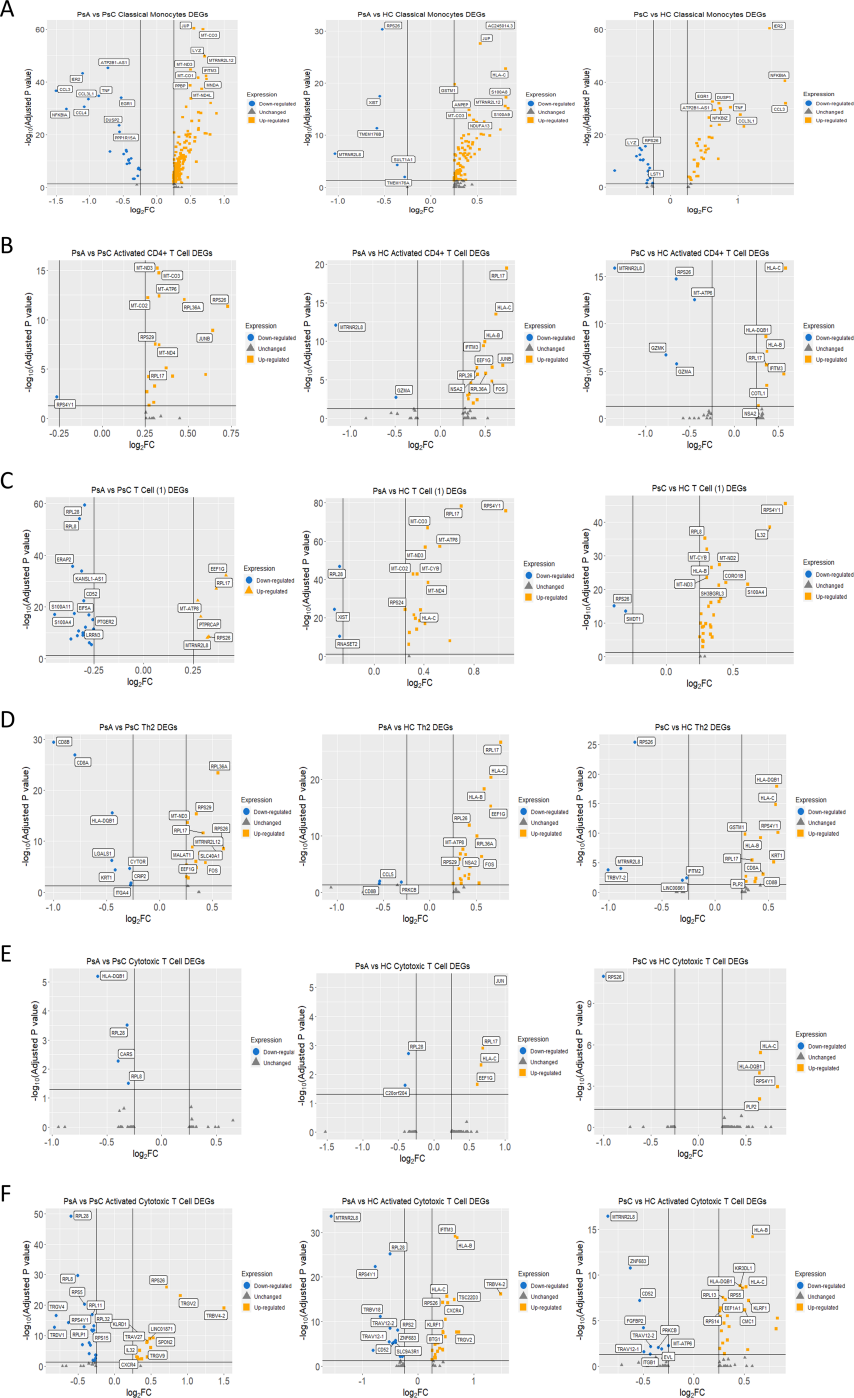
Differentially expressed genes from the scRNA-seq analysis. **(A-F)** Volcano plots comparing between groups by cluster. Genes labeled in yellow were upregulated, and genes labeled in blue were downregulated considering a logFC threshold of |0.25|.

The pro-inflammatory genes, *TNF, IL-1B, CCL3*, and *CCL4*, were upregulated in PsC patients versus HC and PsA (**Figure 2A**). Of note, anti-inflammatory genes *NFKBIA* and *TNFAIP3* were elevated in PsC patients as well. SNPs in both coding and non-coding regions of these genes have been associated with psoriasis through GWAS studies (23). Genes responsive to type-I interferons *MNDA, IFITM3, IFI30*, and *IFI6* were upregulated in PsA compared to both PsC and HC. Pathway enrichment analysis found interferon signaling to be significantly over-represented in the PsA vs PsC comparison only (q-value= 3.15 x10^-3^). Pathways perturbed in PsC patients included NFκB signalling network (PsA vs PsC q-value = 2.69 x10^-7^, PsC vs HC q-value = 5.41 x10^-17^), IL-17 signaling (PsA vs PsC q-value = 1.18 x10^-4^, PsC vs HC q-value = 3.47 x10^-7^) and human cytomegalovirus infection (PsA vs PsC q-value = 1.14 x10^-6^ and PsC vs HC q-value = 4.48 x10^-6^).

#### Activated CD4^+^ T cell

*JUNB* and *FOS*, members of the AP-1 transcription factor family, were upregulated in PsA compared to HC and PsC (**Figure 2B**). The most significant over-represented pathway for PsA patients was nonsense mediated decay (NMD) independent of the exon-exon junction complex (EJC) (PsA vs PsC q-value = 5.49 x10^-9^, PsA vs HC q-value = 1.68 x10^-5^). Ribosomal genes *RPL17, RPL26, RPL36A, RPS17, RPS26, RPS29*, and *RPS4Y1* contributed to this pathway. These genes were upregulated in PsA patients except *RPS4Y1* (LOG2FC= -0.27, p_adj_ = 0.006), which was downregulated when compared to PsC (**Figure 2B**).

#### T Cell (1)

This global T Cell cluster had ribosomal and mitochondrial genes primarily overexpressed in PsA patients when compared to HC (**Figure 2C**) resulting in enrichment of metabolic pathways pertaining to oxidative phosphorylation (PsA vs HC q-value = 9.13 x 10^-8^) and the electron transport chain (q-value = 1.49 x 10^-8^). In the PsA vs PsC comparison, the ribosomal genes contributed to the enrichment of the NMD independent of EJC pathway (q-value = 0.012). The PsC vs HC comparison followed a similar trend, with most pathways that were significantly over-represented being related to translation or metabolic pathways, e.g., electron transport chain (OXPHOS system in mitochondria, q-value = 8.59 x10^-4^).

#### Th2 Cell

The DEGs in the PsA vs PsC comparison were enriched for pathways related to translation, including the NMD independent of EJC pathway (q-value = 3.18 x10^-6^). In the PsA vs PsC comparison, the antigen processing and presentation (q-value = 3.14 x10^-3^), IL12- mediated signaling events (q-value = 1.59 x10^-3^), and CD4 T cell receptor signaling -JNK cascade (q-value = 4.52 x10^-2^) were enriched with the genes *CD8A* (LOG2FC = -0.80, p_adj_ = 1.1 x10^-27^), *CD8B* (LOG2FC = -1.00, p_adj_= 4.06 x10^-30^) and *CD4* (LOG2FC = 0.26, p_adj_ = 5.04 x10^-6^) contributing mainly to the first two pathways. However, only 6.2% of cells in this cluster expressed *CD8A* in PsA patients whereas 37.5% expressed it in PsC. Similar proportions were observed for *CD8B*, expressed by 6.7% of cells in PsA and 35.1% of cells in PsC. In the PsC vs HC comparison the most significant enriched pathways were cell adhesion molecules (CAMs, q-value = 7.84 x10^-7^) and antigen processing and presentation (q-value = 2.17 x10^-6^). Several pathways related to autoimmunity, including: type I diabetes mellitus (q-value = 2.14 x10^-3^), graft-versus-host disease (q-value = 1.85 x10^-3^), allograft rejection (q-value = 1.35 x10^-3^), and autoimmune thyroid disease (q-value = 3.80 x10^-3^) were also enriched. In the PsA vs HC comparison the most significantly enriched pathways were the NMD independent of EJC pathway (q-value = 3.70 x10^-7^) and various pathways related to translation. Enrichment of AP-1 transcription factor network (q-value = 7.58 x10^-3^), TLR signaling (q-value = 3.49 x10^-2^), and TNF signaling (q-value = 4.52 x10^-2^) was found driven by *DUSP1* (LOG2FC = 0.45, p_adj_ = 4.08 x10^-6^), *JUN* (LOG2FC = 0.42, p_adj_ =7.00 x10^-4^), *JUNB* (LOG2FC = 0.53, p_adj_ = 0.02), *FOS* (LOG2FC = 0.56, p_adj_ = 4.99 x10^-7^), and *CCL5* (LOG2FC = -0.54, p_adj_ =9.48x10^-3^) (**Figure 2D**).

#### Cytotoxic T Cell

There was no significantly over-represented pathway for PsA vs HC after Bonferroni correction. In the PsA vs PsC comparison (**Figure 2E**), enriched pathways were related to protein translation, including the NMD independent of the EJC pathway (q-value = 4.62 x10^-2^). In the PsC vs HC (**Figure 2E**) comparison autoimmune thyroid disease (q-value = 7.52 x10^-3^) and type I diabetes mellitus (q-value = 5.12 x10^-3^) were significantly enriched.

#### Activated Cytotoxic T cell

PsA was characterized by significantly upregulated variable beta and alpha chains such as *TRAV27* (LOG2FC = 0.45, p_adj_= 1.69 x10^-8^) and *TRBV4-2* (LOG2FC = 1.50, p_adj_ = 8.43 x10^-20^) when compared to PsC (**Figure 2F**). PsA expression of *TRBV4-2* when compared to HC was elevated as well (LOG2FC = 1.48, p_adj_= 6.23 x10^-17^), suggesting a possible expansion of activated cytotoxic T cells. In both PsC and PsA patients, when compared to HC, *IFITM3* expression was elevated. Pathways that were over-represented in PsA vs HC were the NMD independent of EJC (q-value = 1.17 x10^-3^), translation pathways, and human cytomegalovirus infection (q-value = 3.49 x 10^-2^). The NMD independent of EJC pathway (q-value = 2.69 x10^-9^) was also over-represented in the PsC vs HC comparison. In PsA vs PsC, NMD independent of EJC (q-value = 1.29 x10^-23^) and other pathways related to translation were enriched, including viral mRNA translation (q-value = 6.15 x10^-22^).

### TCR immune profiling

Clonal expansion varied in the 3 groups. PsC patients did not show the same degree of clonal expansion as PsA patients (**Supplementary Figure 2**). To identify the cellular source of the T cell expansions, clonotype information was superimposed onto the cellular clusters. The clonotypes showing a large expansion were primarily restricted to the Activated Cytotoxic T Cell cluster (**Supplementary Figure 3**). Of note, the two highest clonotypes in PSA3 might represent the same hyper expansion in response to antigen (**Supplementary Figure 2C**, **Supplementary Table 2**).

#### Activated Cytotoxic T Cell subclusters

Sub-clustering of the activated cytotoxic T cell cluster was performed using five principal components (**Supplementary Figure 4**). All individuals in the dataset contributed cells to each of the six subclusters (**Supplementary Figure 4B**). Marker genes defining the cellular subtypes included *GZMK, GZMB, GZMH, ZNF683, CD3D, CD8A, CD8B* and *TRGV2* (**Supplementary Figure 4C**). The largest subcluster was defined by having high *GZMK* expression with moderate expression of other granzymes *GZMB* and *GZMH*. The NK cell phenotype subcluster was defined by low expression of *CD3D, CD3G, CD8A* and *CD8B*, but high expression of granzymes *GZMK, GZMB, GZMH* and granulysin (*GNLY*) (**Supplementary Figure 4C**). Clonotype overlap coefficients were high amongst subclusters with low cell numbers (**Supplementary Figure 5B**). The subclusters with the largest clonal expansions were the ZNF683+ and GZMH & GZMB HIGH, TRGV2 LOW subclusters (**Supplementary Figure 5C**).

#### Identifying the Cellular Identity of the top 5 clonotypes

**Figure 3:**
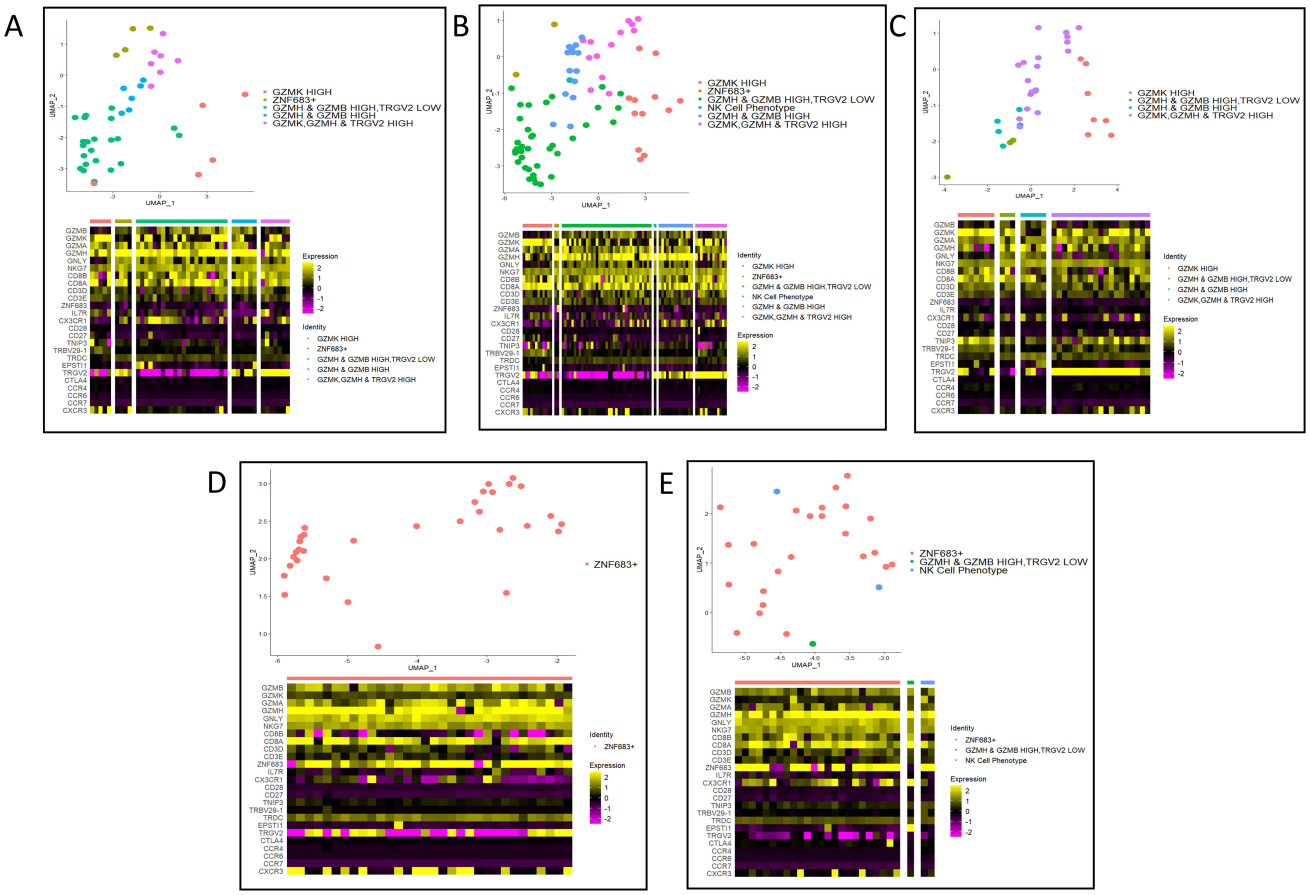
Cellular subcluster identities of the top five expanded clonotypes. **(A)** NA_CASSQDVWGGWSTGELFF clonotype from PSA3. **(B)** CAGGNSGNTPLVF_CASSQDVWGGWSTGELFF clonotype from PSA3. **(C)** CAAPSYNNARLMF_ CASSLDGWDYNEQFF clonotype from PSA1. **(D)** CAVDNFNKFYF_CASSPTMNTEAFF clonotype from HC2. **(E)** CILMDGGATNKLIF_ CASSLEGGTFYTEAFF clonotype from PSA1.

Four of the top five clonotypes were found in PsA patients (**Supplementary Figure 2D**). To assess the cellular identity of these five clonotypes, the amino acid sequence of each clonotype was used to subset the cellular subcluster identities visualized on a UMAP plot (**Figure 3**). Clonotypes from PsA patients were found in cells with heterogeneous transcriptional profiles (**Figures 3A–C, E**). Whereas the clonotype from HC2 was found only in cells of the ZNF683+ subcluster (**Figure 3D**). The two most abundant clonotypes were found in the same patient, across multiple cell subclusters, but primarily in the GZMH & GZMB HIGH, TRGV2 LOW subcluster supporting the notion that these two clonotypes are likely the same and represent a hyper expansion in response to an antigen.

#### NanoString nCounter^®^ DEGs

A custom panel was derived from DEGs identified in the scRNA-seq dataset. A total of 8 HC, 12 PsA and 11 PsC patients were included in the Classical Monocyte experiment. A Kruskal Wallis test comparing the three groups found no gene significantly differentially expressed (p > 0.05) in these cells. In the CD4^+^ T cell assay 8 HC, 13 PsA and 11 PsC patients were included. *C-FOS* (p-value = 0.04), *JUNB* (p-value < 0.01), *NFKBIA* (p-value = 0.01), *CXCL8* (p-value = 0.02), *C-JUN* (p-value = 0.04), and *TNFAIP3* (p-value = 0.01) were significantly DEGs according to a Kruskal Wallis test (**Supplementary Figure 6**). In the CD8^+^ T cell assay 6 HC, 13 PsA and 11 PsC patients were included. Four genes were DEGs across the three groups: *TNFAIP3* (p-value = 0.03), *C-JUN* (p-value = 0.04), *JUNB* (p-value = 0.01) and *NFKBIA* (p-value = 0.04) (**Supplementary Figure 7**).

#### Immunoblotting of IκBα and NFKBIA transcript levels

**Figure 4:**
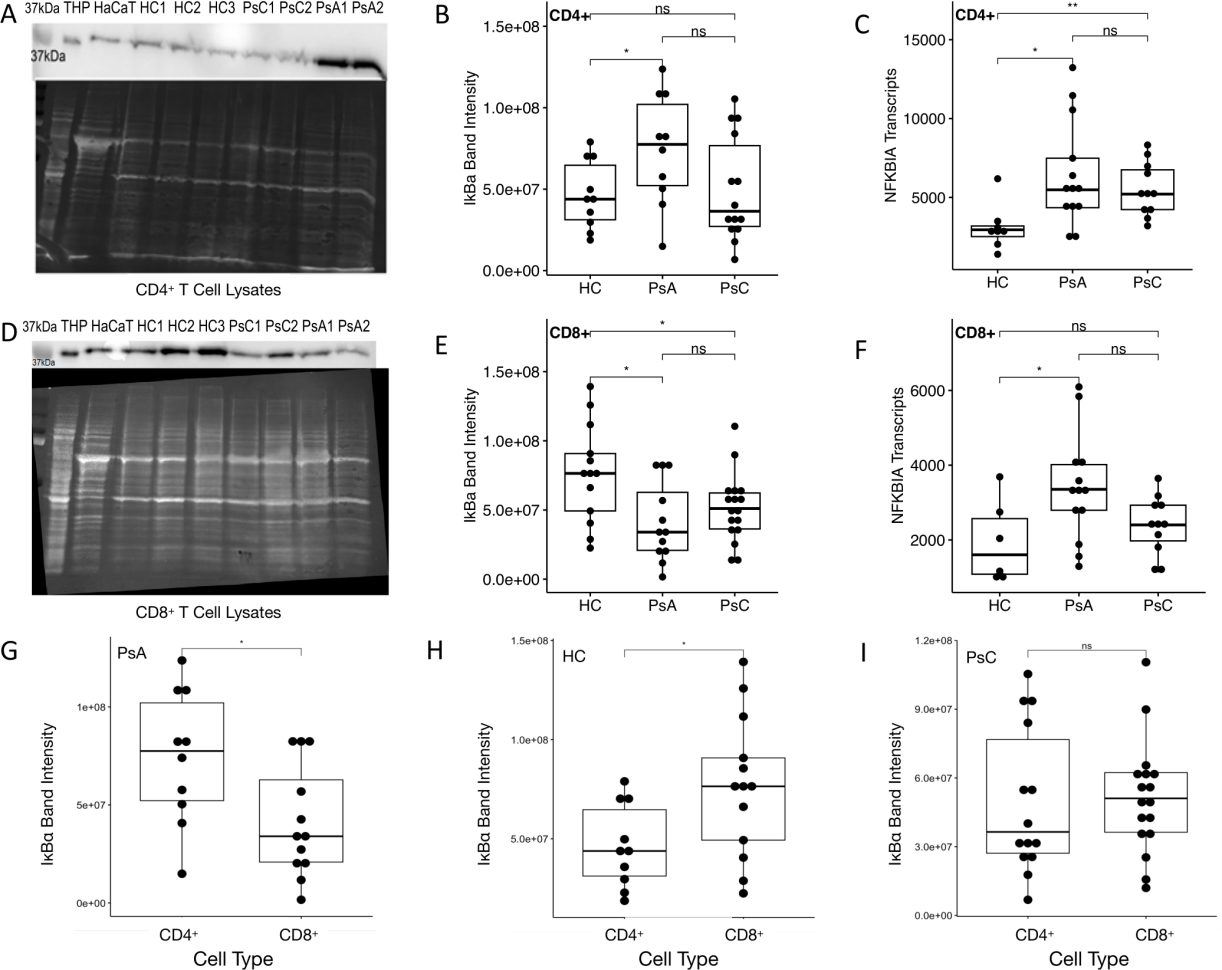
IκBα and *NFKBIA* expression. Representative immunoblot, IκBα band intensities and *NFKBIA* transcript levels in CD4^+^ T cell lysates **(A-C)** and CD8^+^ T cell lysates **(D-F)**, respectively). Comparison of IκBα band intensities in CD4^+^ T and CD8^+^ T cell lysates in **(G)** PsA patients, **(H)** healthy controls and **(I)** PsC patients. THP and HaCaT cell lysates are used as positive controls. ns non-significant, * p-value < 0.05 & ** p-value <0.01 according to a pairwise Wilcoxon Rank Sum test.

10 HC, 11 PsA and 12 PsC patients were included in the CD4^+^ immunoblotting assays, while 13 HC, 12 PsA and 17 PsC patients were included in the CD8^+^ assays. In CD4^+^ T cell lysates PsA patients had higher relative levels of IκBα compared to HC and PsC patients (**Figures 4A, B**). PsA patients also had higher *NFKBIA* transcript levels than HC in CD4^+^ T- cells (**Figure 4C**). In CD8^+^ T cell lysates PsA patients had significantly lower levels of IκBα than HC (**Figures 4D, E**). PsC patients also had lower levels of IκBα in CD8^+^ T cell lysates compared to HC (**Figure 4E**). This contrasts with the *NFKBIA* transcript levels, which were higher in PsA compared to HC (**Figure 4F**). IκBα levels in CD4^+^ T cell lysates compared to IκBα levels in CD8^+^ T cell lysates in PsA patients were higher (**Figure 4G**). This was discordant to the pattern observed in HC, where CD8^+^ T cell lysates had higher IκBα levels than CD4^+^ T cell lysates (**Figure 4H**). Whereas IkBa levels were similar in both cell types in the PsC cohort (**Figure 4I**).

#### Immunoblotting of A20 and TNFAIP3 transcript levels

**Figure 5:**
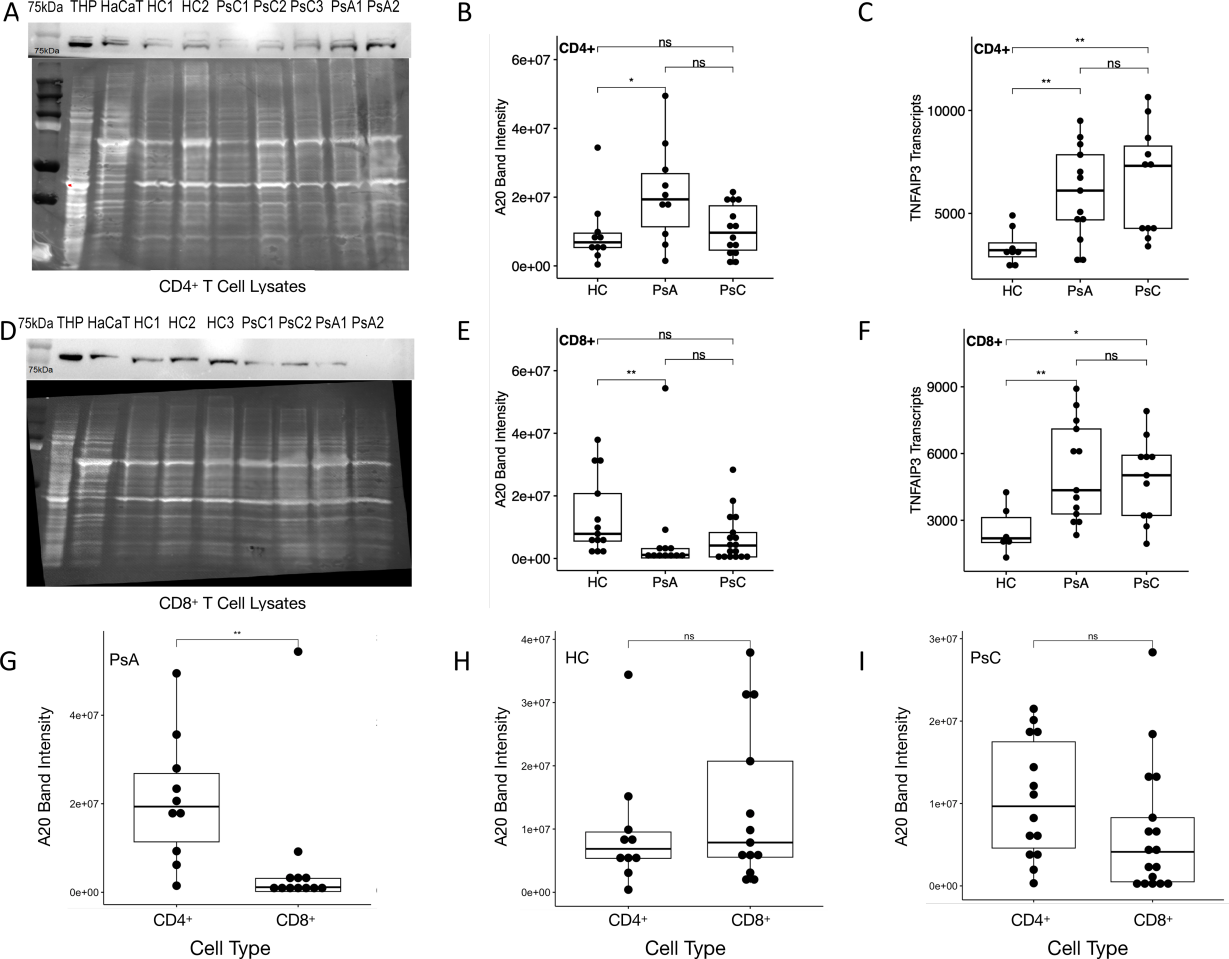
A20 and *TNFAIP3* expression. Representative immunoblot, A20 band intensities and *TNFAIP3* transcript levels in CD4^+^ T cell lysates **(A-C)** and CD8^+^ T cell lysates **(D-F)**, respectively). Comparison of A20 band intensities in CD4^+^ T and CD8^+^ T cell lysates in **(G)** PsA patients, **(H)** healthy controls and **(I)** PsC patients. THP and HaCaT cell lysates are used as positive controls. ns non-significant, * p-value < 0.05 & ** p-value <0.01 according to a pairwise Wilcoxon rank sum test.

10 HC, 11 PsA and 12 PsC patients were included in the CD4^+^ immunoblotting assays, while 13 HC, 12 PsA and 17 PsC patients were included in the CD8^+^ assays. In PsA CD4^+^ T cell lysates, A20 levels were higher compared to HC (**Figures 5A, B**). PsA patients had higher *TNFAIP3* transcript levels than HC in CD4^+^ T cells (**Figure 5C**). In CD8^+^ T cell lysates the expression of A20 was lower in PsA patients compared to HC (**Figures 5D, E**). *TNFAIP3* levels in CD8^+^ T cells were higher in PsA compared to HC (**Figure 5F**). PsA patients had lower A20 levels in CD8^+^ T cell lysates than CD4^+^ T cell lysates (**Figure 5G**). A20 levels in CD4^+^ and CD8^+^ T cell lysates in HC and PsC were similar (**Figures 5H, I**). A20 was decreased in PsA CD8^+^ T cell lysates compared to PsA CD4^+^ T cell lysates (**Supplementary Figure 8**).

## Discussion

Here, we harnessed scRNA-seq to identify potential susceptibility factors for psoriatic disease. Of note, a previous single-cell sequencing report found significant deregulation of ribosomal protein genes in PsA patients when compared to psoriasis across cell types, and deregulation of AP-1 transcription factors in T cell and NK cell clusters. These findings are partially supported by our own observations in the Activated CD4^+^ T cell cluster. The role of classical monocytes in psoriasis pathology is largely unknown. We observed monocyte DEGs were enriched for pathways related to TNF, Interferon, NFκB and IL-17 signaling as well as osteoclast differentiation. All these pathways have previously been associated with psoriatic disease (24–27). Polymorphisms in the *TNF* gene, specifically the 238 (G➔A) variant, are risk factors for PsA (28). *TNF* and other pro-inflammatory genes such as *CCL3, CCL4*, and *IL1B* were upregulated in PsC patients compared to PsA and HC. However, these genes are generally associated with intermediate monocytes (29). On the other hand, anti-inflammatory genes *NFKBIA* and *TNFAIP3* were elevated in PsC patients as well. We sought to validate the DEGs identified in the Classical Monocyte cluster on the NanoString nCounter^®^ platform but could not replicate the findings in an independent cohort. This was possibly due to our scRNA-seq DEGs being driven by one individual in the PsC group of our scRNA-seq dataset, which may have been a result of TLR stimulation (30) in the Classical Monocyte cluster (**Supplemntary Figure 8**). However, given the interesting set of DEGs in the monocyte cluster, we decided to investigate them in a separate cohort of patients in CD8^+^ and CD4^+^ T cells using a custom NanoString nCounter^®^ gene panel.

At the bulk RNA level PsA patients had upregulated *TNFAIP3* and *NFKBIA* compared to HC in both CD4^+^ and CD8^+^ T cells. PsC patients showed the same upregulation in CD4^+^ and CD8^+^ T cells for *TNFAIP3*, while *NFKBIA* was upregulated only in CD4^+^ cells compared to HC. Both genes encode proteins, A20 and IκBα respectively, that can negatively regulate inflammation (31, 32). *TNFAIP3* transcripts are lower in lesional epidermis of psoriasis patients than in uninvolved epidermis, and that of HC, indicating an inflammatory state in keratinocytes (33). In contrast to our findings, others have shown that *TNFAIP3* transcripts were lower in whole blood of psoriasis patients compared to healthy controls, and that A20 levels were lower in psoriatic skin biopsies compared to HC skin (34). In our dataset, PsA patients showed higher expression of A20 in CD4^+^ T cells compared to both HC and PsC, which is consistent with the nCounter^®^ data, where PsA patients only had elevated *TNFAIP3* transcripts compared to HC. However, CD8^+^ T cell PsA lysates had lower expression of A20 compared to HC, which contrasts the nCounter^®^ findings. This may suggest that *TNFAIP3* transcripts are not being translated into A20 in PsA CD8^+^ T cells or that the protein is being degraded.

Translational blocking in CD8^+^ T cells could be due to miRNA silencing. One of many miRNAs that target *TNFAIP3* is miR-125b-5p, which was recently found to be an important regulator of IL-1B induced inflammation in synovial osteochondrocytes in osteoarthritis (35). miRNA sequencing experiments of isolated cell subsets from PsA patients would be needed to clarify if *TNFAIP3* translation is repressed by miRNA. Alternatively, translation of *TNFAIP3* may be reduced due to a dysfunctional NMD pathway. The NMD independent of the EJC pathway was the most significantly enriched pathway in our scRNA-seq dataset across all clusters and pairwise comparisons, largely attributed to differential expression of ribosomal protein genes. Recently, increased expression of ribosomal proteins in PBMCs of PsA patients was identified when compared to psoriasis and healthy controls (36). Cell specific NMD pathway dysfunction in mice produces a variety of disease phenotypes (37). No canonical member of the NMD pathway was deregulated in our scRNA-seq dataset, however the ribosomal proteins that make up the UPF1:eRF3 complex on translated mRNA in the NMD pathway were altered at the transcript level (38). A dysfunctional 60s or 40s ribosomal unit may lead to aberrant activity of the UPF1:erf3 complex and subsequently lead to translation inhibition. Measuring phosphorylated UPF1 in CD8^+^ T cells of PsA patients would enable assessment of NMD pathway activity since phosphorylation of UPF1 precedes transcript degradation but is intimately involved in translational repression (39). Interestingly, in a study that tried to classify psoriasis patients, healthy controls and PsA patients using single-cell transcriptomics and cell surface epitopes, differential expression of both mitochondrial (*MT-CO3, MT-ND1, MT-ND3*) and ribosomal protein (*RPS26*) genes were detected across most cell types when comparing PsA to psoriasis or healthy controls (15).

IκBα also exhibited a similar pattern of expression to A20: elevated *NFKBIA* transcripts in PsA CD8^+^ T cells and decreased IκBα in PsA CD8^+^ T cells. As previously mentioned, translation inhibition and even transcript degradation could be due to aberrant miRNA activity in CD8^+^ T cells by miR-300 or four other miRNAs that target *NFKBIA* – miR-381-3p, miR-624-5p, miR-4687-3p and miR-6802-3p (40).

The T cell immune profiling data provided evidence of possible antigenic selection occurring in PsA. Notably all expanded clonotypes were observed in the activated cytotoxic T cell cluster. This finding is consistent with a recent study that performed scRNA-seq on paired PsA synovial fluid and peripheral blood showing that pronounced T cell clonal expansions were CD8^+^ T cells and were more expanded in the synovial fluid than in the peripheral blood (13). They did not, however, use a control cohort to compare the PsA T cell expansions observed in the peripheral blood. We observed varying transcriptional states among activated cytotoxic T cells within the top five clonotypes in our dataset.

The one HC expansion was characterized by a *ZNF683* ^+^ phenotype whereas PsA was characterized by most cells having different types and degrees of granzyme gene expression. ZNF683^+^ CD8^+^ T cells are considered long lived effector-type T cells that are essential in combating CMV infection (41). CMV infects about 50% of all adults by the age of 40 and generally remains latent in myeloid cells throughout one’s lifetime, and these T cells have been shown to combat the CMV reactivation phase (42). The high expression of *GZMB* in the expanded PsA CD8^+^ T cells is indicative of effector cytotoxic T cells as GZMB exists in cytotoxic granules and is used to kill virally infected cells (43). In our dataset we found two clonotypes which likely represent the same expansion, making up 10% of the TCR repertoire for one PsA patient. This clonotype was found across effector-like cells characterized broadly by high *GZMH* and *GZMB* expression. HLA-typing information on this patient allowed us to predict antigen specificity using the VDJdb database (22). The predicted epitope was IE1 from CMV. Psoriasis is more severe in CMV seropositive individuals (44). In PsA, CMV DNA was found to be present in 3/6 synovial tissues analyzed by DNA *in situ* hybridization (45). Two of six specimens demonstrated CMV early antigen positivity indicative of a replicative state of the CMV virus (45). Recently, a review reported that CMV likely plays a role in the inflammation status of RA but is protective of bone erosions (46). CMV has been proposed as a potential trigger of multifactorial components that ultimately lead to the onset of PsA and autoimmune diseases.

One of the limitations of this study is the small sample size of the scRNA-seq and Nanostring dataset. PsA and PsC patients selected for scRNA-seq had low disease activity. Patients with higher disease activity could provide better signals associated with disease. However, PsA patients with the highest disease activity are more likely to be on systemic biological therapies, which alters genetic signatures across whole blood in RA patients (47), and likely does the same in PsA patients making them ineligible for studies of this nature. Additionally, patients were matched for the scRNA-seq dataset, but not for the NanoString nCounter^®^ experiment. Age was significantly different among the three groups. Another limitation is potential imperfect isolation of CD4^+^ and CD8^+^ T cells in our nCounter^®^ experiment, which may obscure expression patterns unique to each cell type. All these factors may have contributed to the significant DEGs being identified in the experiments.

Further studies may include patients with PsC prior to development of PsA including collection of PBMCs over time, since in most cases psoriasis precedes the onset of PsA (48). Capturing the biological changes of PBMCs in a PsC to PsA conversion would help elucidate the etiology of disease. However, this type of study is viable but logistically difficult due to the average annual conversion rate of PsC patients to PsA being only 2.7% (49).

Given that *TNFAIP3* and *NFKBIA* are important in regulating inflammation and our study demonstrates translational inhibition, it would be interesting to explore if inhibiting these inflammation pathways are viable therapeutic options for PsA (32, 50). These results contribute to a growing body of literature linking CD8^+^ Cytotoxic T-cells to the pathogenesis of PsA, which is regarded as an MHC Class I mediated disease (13, 51, 52).

## Data Availability

The original contributions presented in the study are included in the article/**Supplementary Material.** Further inquiries can be directed to the corresponding author/s.
