# Supplementary material for: Single-cell RNA sequencing of circulating immune cells supports inhibition of TNFAIP3 and NFKBIA translation as psoriatic arthritis biomarkers
Source: Front Immunol. 2025 Feb 7;16:1483393. doi: 10.3389/fimmu.2025.1483393 (PMC11842318; doi:10.3389/fimmu.2025.1483393)
Supplement: Supplementary file 1 [file DataSheet1.docx]

**Supplementary Results**

**Annotation of scRNA-seq dataset**

The Seurat function ‘FindAllMarkers’ was used to identify cell type markers. The expression of a particular gene in all cells in a cluster of interest was compared against all other clusters using a Wilcoxon rank sum test. In total 18 cell clusters were identified. The ‘T Cell w/T-regs’ cluster was heterogeneous containing multiple T-cell populations, it was defined by having high *CD3E, MAL, CCR7, LEF1, CD3D, IL-7 R, GATA3* and *FOXP3* compared to all other clusters in the dataset. High expression of *CD3D* and *CD3E* was used to label T Cell (1) cluster. High expression of *CD14, S100A9, S100A8*, and low expression of *FCGR3A* characterized the Classical Monocyte cell cluster. NK Cells were defined by having high *NKG7, KLRD1, GNLY*, moderate *FCG3RA* expression and low to no *CD3D* or *CD3E* expression. Th2 Cells were characterized by high *CD3D, CD3E, IL-7R, IL-32*, and *GATA3*. Activated CD4+ T cells were labeled based on high *CD3D, CD3E, RORA* and low *CCR7* expression. Activated cytotoxic T cells were defined by high expression of *CD3E*, *CD3D*, *NKG7*, *CD8B*, and *CD8A*. Immature B Cells were characterized by high expression of *CD79A, TCL1A, IGHM, MS4A1*, and *CD19*. The Non-classical Monocyte cluster was defined by low *S100A8, S100A9, LYZ*, and high *FCGR3A*, *AIF1*, *MS4A7* and *CD68* expression. Cytotoxic T cells were defined by high expression of *CD3D, CD3E, CD8A, CD8B* and low *NKG7* expression. Innate lymphoid cells (ILCs) are difficult to define as they share expression of many molecules with T cell subsets and can express CD3 at the mRNA level. The ‘findallmarkers’ function for the ILCs cluster did not give any marker of particular importance, as genes that were found to be differentially expressed in this cluster compared to all other cells were indiscriminately expressed, making it difficult to discern a cellular identity for this cluster. When compared to a T-Cell cluster, T- Cell (1), it became apparent that this cluster expressed *CD3E* and *CD2* at low levels. *RORA* was expressed highly, but not in all cells pertaining to this cluster, and *KLRD1* and *NKG7* expression was detected. Mature B Cells were defined by high *CD79A*, *MS4A1*, *CD19* and an absence of *TCL1A* expression. The Megakaryocyte Progenitor Cell was defined by high *PPBP*, *PF4* and *CAVIN2* expression. The NK Cell (2) cluster was defined by high *NKG7*, and *GNLY* expression, albeit lower than NK Cell (1). There is likely ILCs present in this cluster considering the expression of *TBX21*, *GATA3* and *CD2* being relatively high in this cluster. The dendritic cell cluster was defined by high *VCAN*, *CD68*, *FCER1A*, *CLEC10A, CD1C, ENHO* and low *CD14* and *FCGR3A* expression. Plasmacytoid dendritic cells were defined by high *GZMB, TCF4, LILRA4, PLD4*, and *SERPINF1* expression. The Erythroid Precursor Cell was defined by high *MCM7, MCM2, RRM2, GINS2, PCLAF, CLSPN, DTL, TYMS*, and *MKI67* expression. Due to the small size of this cluster the list of marker genes generated by ‘FindAllMarkers’ were input into PanglaoDB (v.27/03/2020)[1]. The top score for this cell marker list according to PanglaoDB was ‘Unknown’, the second top score was ‘Germ Cells’ which is not applicable to PBMCs; therefore, the third highest scoring label was assigned. The final cell cluster in this dataset was the Circulating Precursor Cells, defined by having high *CD34* expression (Supplementary Figure 1).

**References**

1. Franzén, O, Gan L-M, Björkegren JLM. PanglaoDB: a web server for exploration of mouse and human single-cell RNA sequencing data. Database 2019;2019:baz046.
