# Supplementary figures and images for: Single-cell RNA sequencing of circulating immune cells supports inhibition of TNFAIP3 and NFKBIA translation as psoriatic arthritis biomarkers
Source: Front Immunol. 2025 Feb 7;16:1483393. doi: 10.3389/fimmu.2025.1483393 (PMC11842318; doi:10.3389/fimmu.2025.1483393)

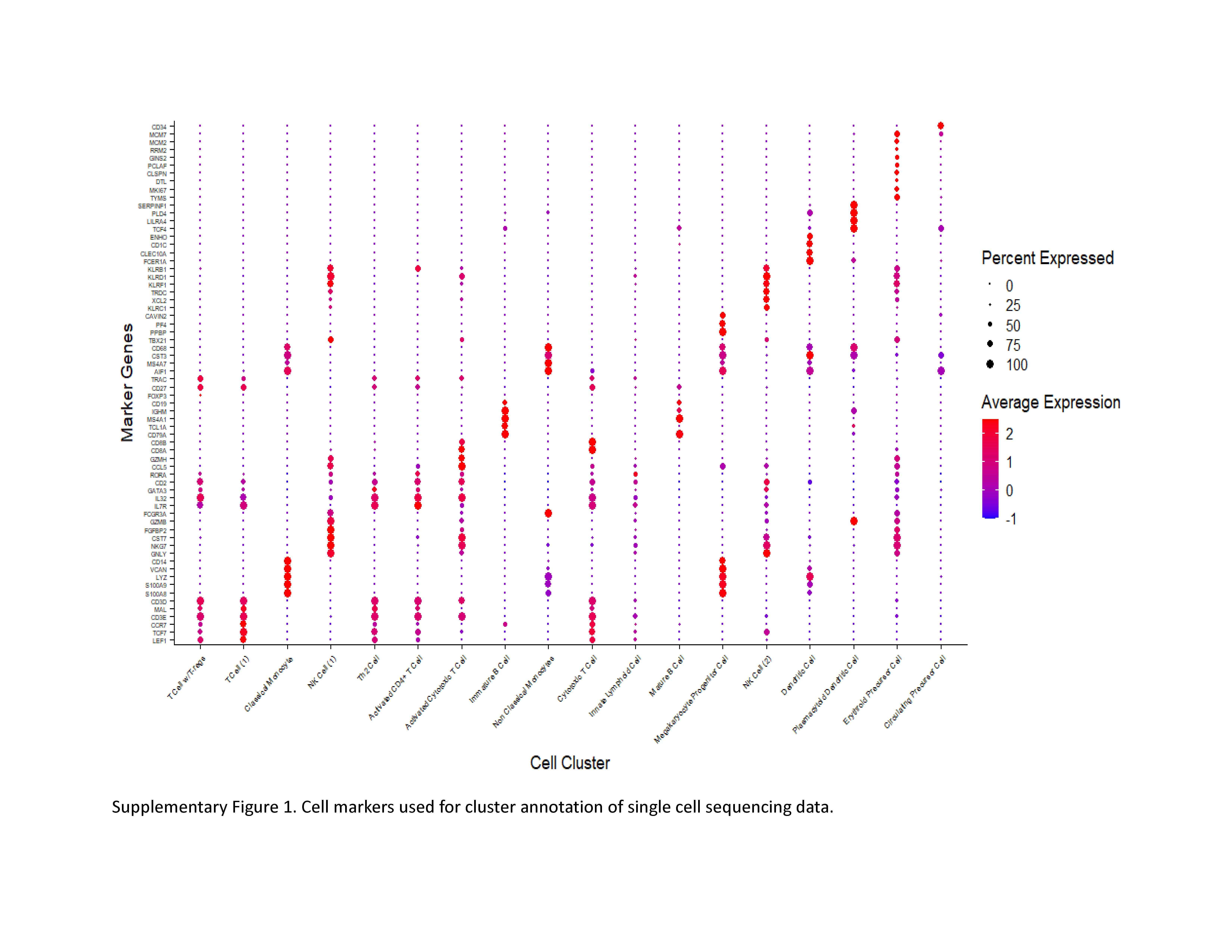

Supplement: Supplementary file 2 [file Image1.tiff]

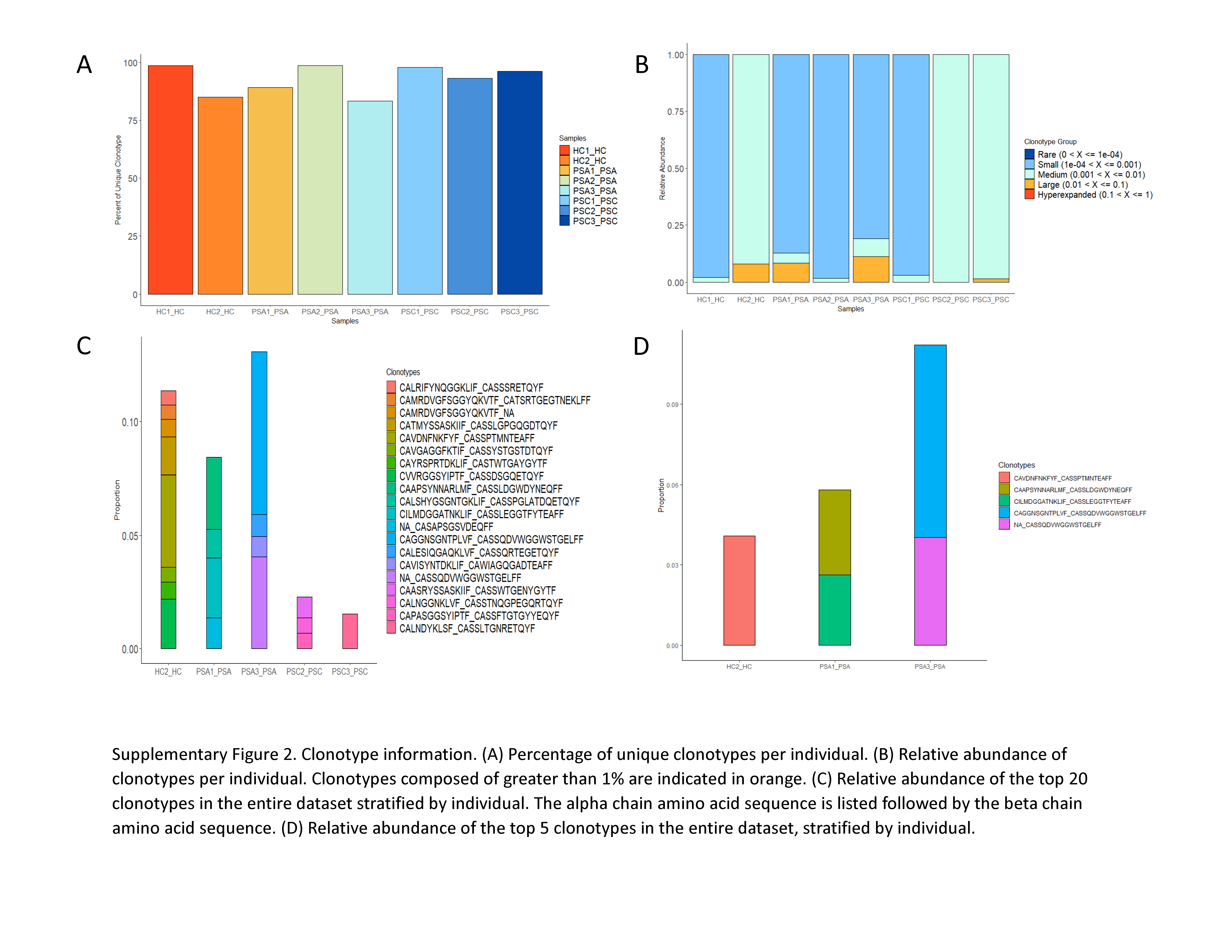

Supplement: Supplementary file 3 [file Image2.tif]

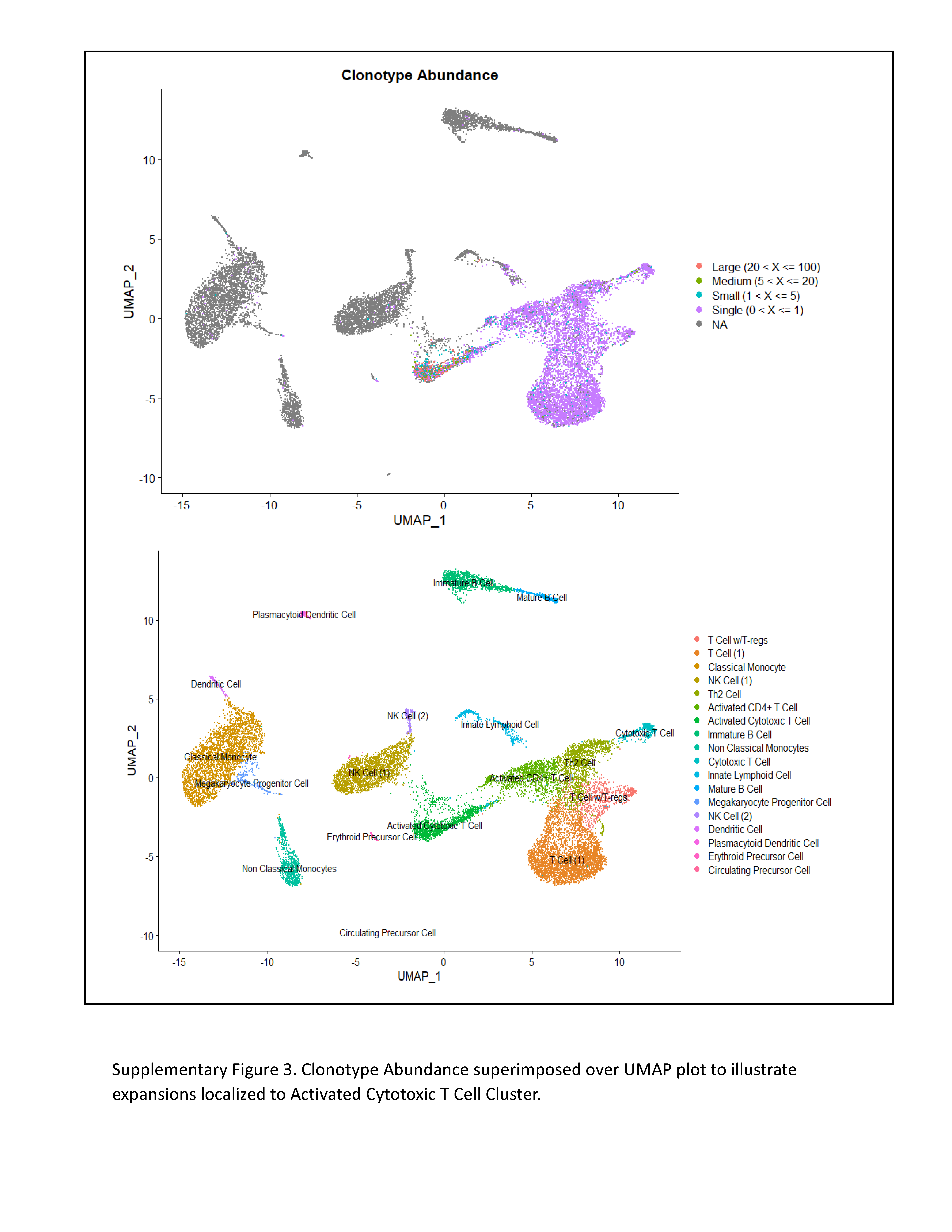

Supplement: Supplementary file 4 [file Image3.tif]

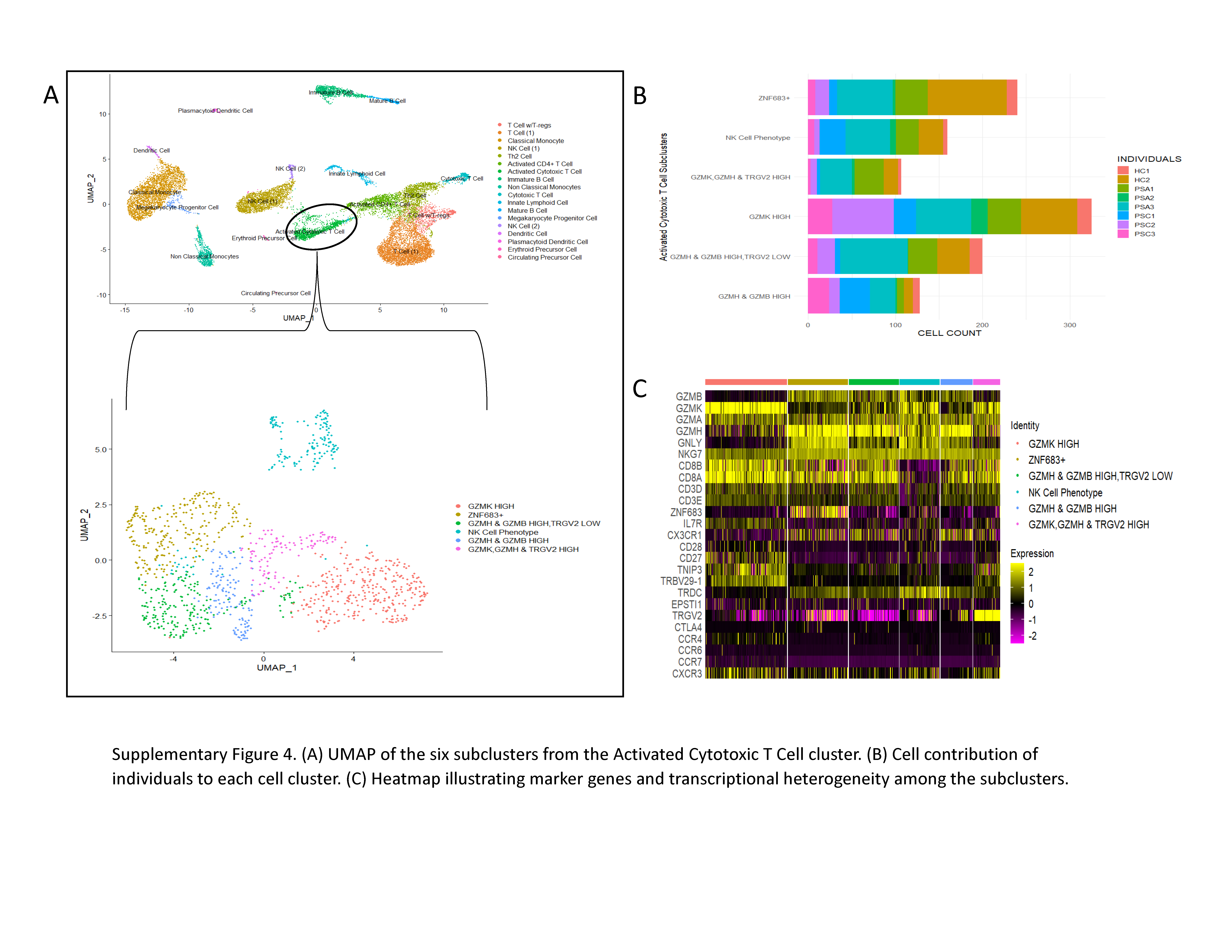

Supplement: Supplementary file 5 [file Image4.tif]

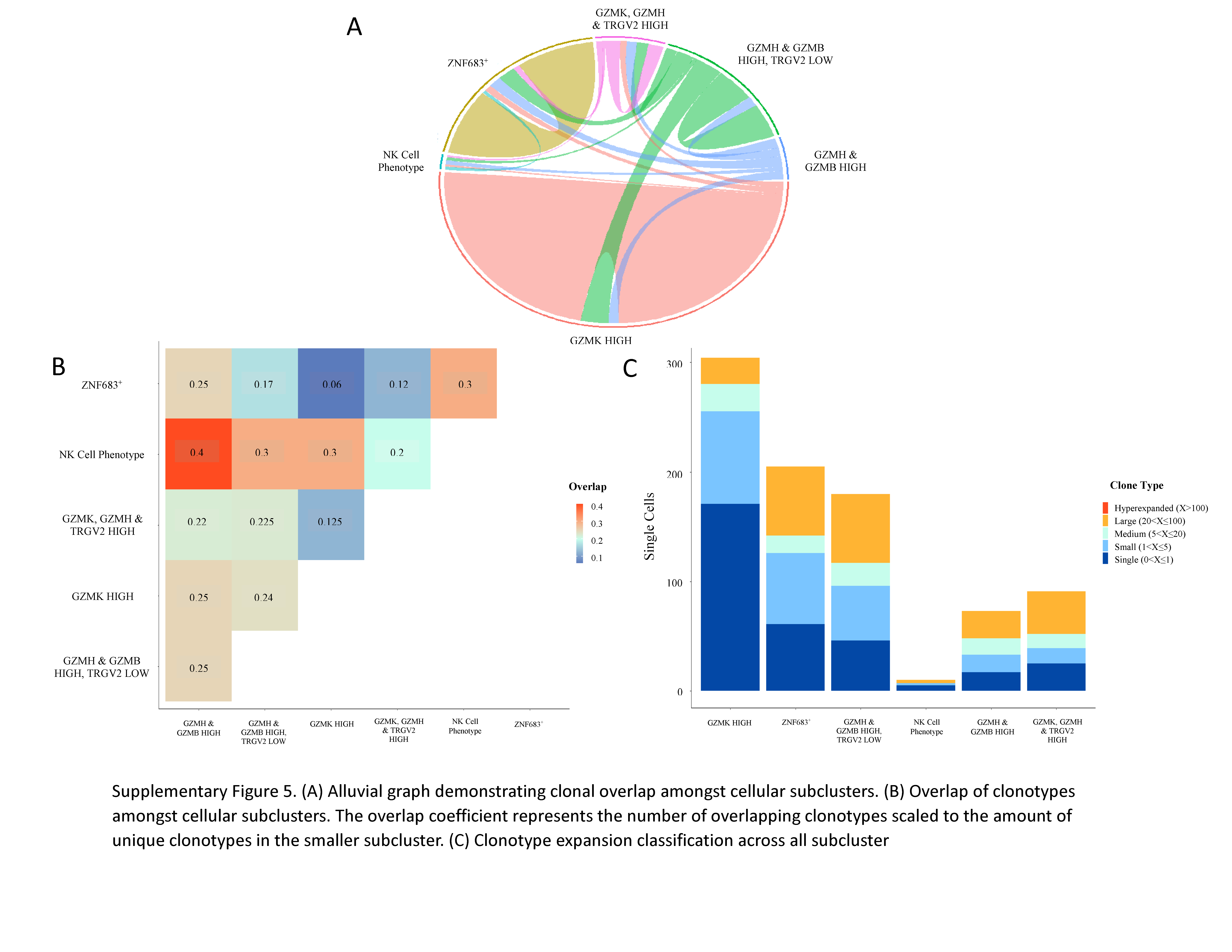

Supplement: Supplementary file 6 [file Image5.tiff]

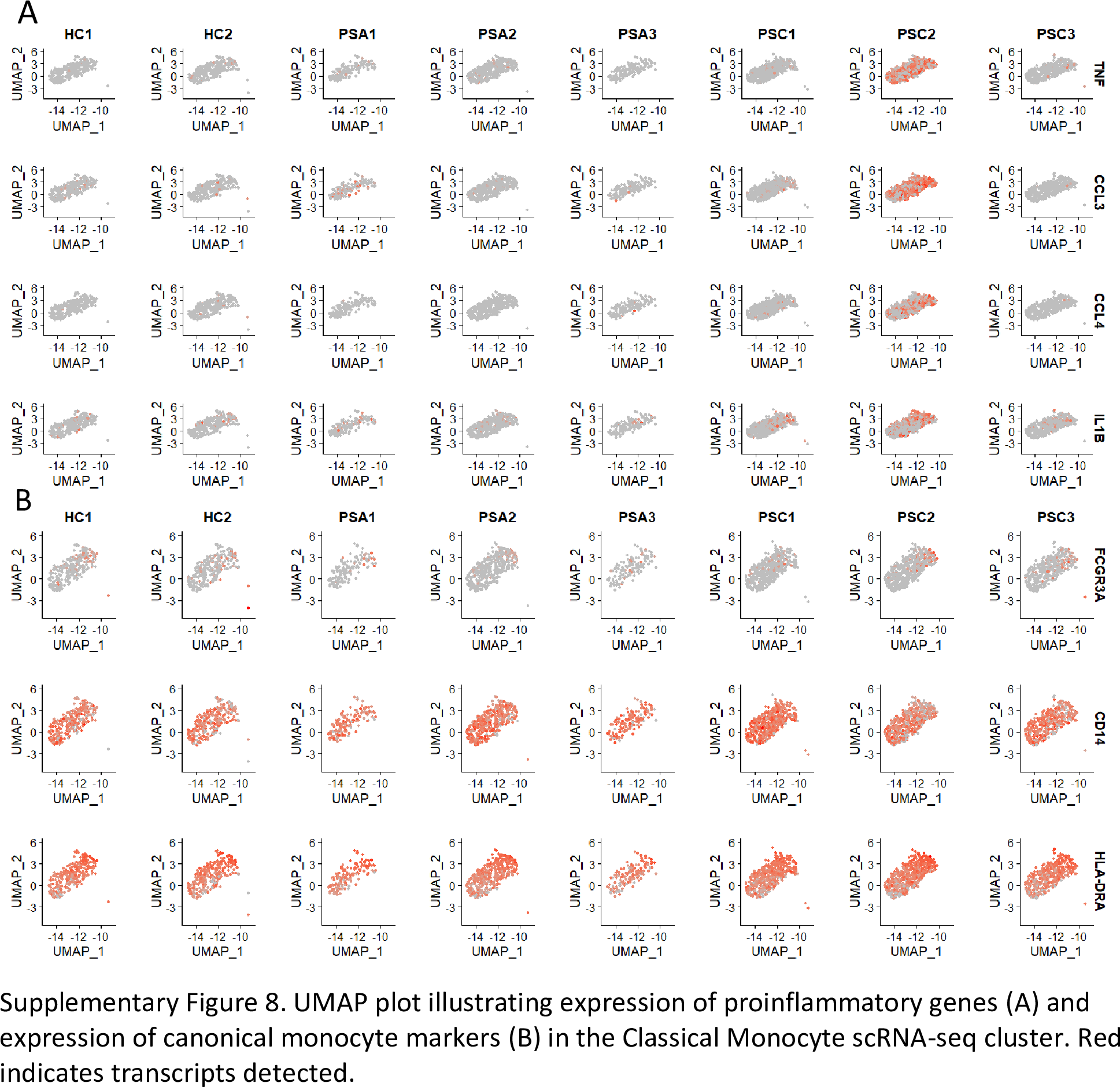

Supplement: Supplementary file 9 [file Image8.tiff]
