# Supplementary material for: Single-cell RNA sequencing of circulating immune cells supports inhibition of TNFAIP3 and NFKBIA translation as psoriatic arthritis biomarkers
Source: Front Immunol. 2025 Feb 7;16:1483393. doi: 10.3389/fimmu.2025.1483393 (PMC11842318; doi:10.3389/fimmu.2025.1483393)
Supplement: Supplementary file 10 [file Table1.docx]

| **Number** | **Gene Name** | **Accession Number** | **Position** | **Target Probe** |
| --- | --- | --- | --- | --- |
| 1 | *ACTB* | NM_001101.2 | 1011-1110 | TGCAGAAGGAGATCACTGCCCTGGCACCCAGCACAATGAAGATCAAGATCATTGCTCCTCCTGAGCGCAAGTACTCCGTGTGGATCGGCGGCTCCATCCT |
| *2* | *B2M* | NM_004048.2 | 236-335 | TACTGAAGAATGGAGAGAGAATTGAAAAAGTGGAGCATTCAGACTTGTCTTTCAGCAAGGACTGGTCTTTCTATCTCTTGTACTACACTGAATTCACCCC |
| *3* | *CCL3* | NM_002983.2 | 682-781 | CTGTGTAGGCAGTCATGGCACCAAAGCCACCAGACTGACAAATGTGTATCGGATGCTTTTGTTCAGGGCTGTGATCGGCCTGGGGAAATAATAAAGATGC |
| 4 | *CCL4* | NM_002984.2 | 202-301 | GAAGCTTCCTCGCAACTTTGTGGTAGATTACTATGAGACCAGCAGCCTCTGCTCCCAGCCAGCTGTGGTATTCCAAACCAAAAGAAGCAAGCAAGTCTGT |
| *5* | *CD86* | NM_175862.3 | 1266-1365 | CCAGCTCTGCTCCGTATGCCAAGAGGAGACTTTAATTCTCTTACTGCTTCTTTTCACTTCAGAGCACACTTATGGGCCAAGCCCAGCTTAATGGCTCATG |
| *6* | *CX3CR1* | NM_001337.3 | 1041-1140 | GGGCGCTCAGTCCACGTTGATTTCTCCTCATCTGAATCACAAAGGAGCAGGCATGGAAGTGTTCTGAGCAGCAATTTTACTTACCACACGAGTGATGGAG |
| 7 | *CXCL10* | NM_001565.2 | 462-561 | GCCATAATTGTTCTTAGTTTGCAGTTACACTAAAAGGTGACCAATGATGGTCACCAAATCAGCTGCTACTACTCCTGTAGGAAGGTTAATGTTCATCATC |
| *8* | *CXCL8* | NM_000584.2 | 26-125 | ACAGCAGAGCACACAAGCTTCTAGGACAAGAGCCAGGAAGAAACCACCGGAAGGAACCATCTCACTGTGTGTAAACATGACTTCCAAGCTGGCCGTGGCT |
| *9* | *CXCR3* | NM_001504.1 | 81-180 | GTGAGTGACCACCAAGTGCTAAATGACGCCGAGGTTGCCGCCCTCCTGGAGAACTTCAGCTCTTCCTATGACTATGGAGAAAACGAGAGTGACTCGTGCT |
| 10 | *DDX58* | NM_014314.3 | 1390-1489 | CGGTTGGTGTTGGGGATGCCAAAAACACAGATGAAGCCTTGGATTATATCTGCAAGCTGTGTGCTTCTCTTGATGCGTCAGTGATAGCAACAGTCAAACA |
| *11* | *C-FOS* | NM_005252.4 | 1462-1561 | GCATCCATGTGTGGACTCAAGTCCTTACCTCTTCCGGAGATGTAGCAAAACGCATGGAGTGTGTATTGTTCCCAGTGACACTTCAGAGAGCTGGTAGTTA |
| *12* | *FOSB* | NM_006732.1 | 3201-3300 | ATATATGGATGTGTGTGTGTGCGTGCGCGTGAGTGTGTGAGCGCTTCTGCAGCCTCGGCCTAGGTCACGTTGGCCCTCAAAGCGAGCCGTTGAATTGGAA |
| 13 | *GAPDH* | NM_001256799.1 | 387-486 | GAACGGGAAGCTTGTCATCAATGGAAATCCCATCACCATCTTCCAGGAGCGAGATCCCTCCAAAATCAAGTGGGGCGATGCTGGCGCTGAGTACGTCGTG |
| *14* | *HLA-A* | XR_430999.1 | 1125-1224 | AGCCTGGGCCCTGTGTGCCAGCACTTACTCTTTTGTAAAGCACCTGTTAAAATGAAGGACAGATTTATCACCTTGATTACAGCGGTGATGGGACCTGATC |
| *15* | *HLA-B* | NM_005514.6 | 938-1037 | CCCTGAGATGGGAGCCGTCTTCCCAGTCCACCGTCCCCATCGTGGGCATTGTTGCTGGCCTGGCTGTCCTAGCAGTTGTGGTCATCGGAGCTGTGGTCGC |
| 16 | *HLA-C* | NM_002117.4 | 896-995 | AGCTGGGAGCCATCTTCCCAGCCCACCATCCCCATCATGGGCATCGTTGCTGGCCTGGCTGTCCTGGTTGTCCTAGCTGTCCTTGGAGCTGTGGTCACCG |
| *17* | *HLA-DQB1* | NM_001243962.1 | 1291-1390 | CCCAGCCAACCTGTGCCCAGAAGGAGGGTTGTACCTTGAAAAGACACTGAAAGAATTTGGGGTGCAAAGTCATGGTGGGCAGAGGAGGTAGAAAATCAAC |
| *18* | *IFI30* | NM_006332.3 | 386-485 | CAGGAACAAAATGTCAGTGGCAGGTGGGAGTTCAAGTGCCAGCATGGAGAAGAGGAGTGCAAATTCAACAAGGTGGAGGCCTGCGTGTTGGATGAACTTG |
| 19 | *IFI6* | NM_002038.3 | 433-532 | AGCAGCGTCGTCATAGGTAATATTGGTGCCCTGATGGGCTACGCCACCCACAAGTATCTCGATAGTGAGGAGGATGAGGAGTAGCCAGCAGCTCCCAGAA |
| *20* | *IFIH1* | NM_022168.4 | 3137-3236 | CCATGTAATTGAGAAAATGCATCACGTCAATATGACCCCAGAATTCAAGGAACTTTACATTGTAAGAGAAAACAAAGCACTGCAAAAGAAGTGTGCCGAC |
| *21* | *IFITM2* | NM_006435.2 | 5-104 | AAACTGTTGAGAAAACGGAACTACTGGGGAAAGGGAGGGCTCACTGAGAACCATCCCGGTAACCCGATCACCGCTGGTCACCATGAACCACATTGTGCAA |
| 22 | *IFITM3* | NM_021034.2 | 478-577 | TGCTGATCTTCCAGGCCTATGGATAGATCAGGAGGCATCACTGAGGCCAGGAGCTCTGCCCATGACCTGTATCCCACGTACTCCAACTTCCATTCCTCGC |
| *23* | *IFNA1* | NM_024013.3 | 110-209 | TCAGCTGCAAGTCAAGCTGCTCTCTGGGCTGTGATCTCCCTGAGACCCACAGCCTGGATAACAGGAGGACCTTGATGCTCCTGGCACAAATGAGCAGAAT |
| *24* | *IFNAR I* | NM_000629.2 | 217-316 | GCCGCAGGTGGAAAAAATCTAAAATCTCCTCAAAAAGTAGAGGTCGACATCATAGATGACAACTTTATCCTGAGGTGGAACAGGAGCGATGAGTCTGTCG |
| 25 | *IFNAR2* | NM_000874.5 | 1438-1537 | TTTCATAACTTTTTAGCCTGGCCATTTCCTAACCTGCCACCGTTGGAAGCCATGGATATGGTGGAGGTCATTTACATCAACAGAAAGAAGAAAGTGTGGG |
| *26* | *IFNB1* | NM_002176.2 | 611-710 | ACAGACTTACAGGTTACCTCCGAAACTGAAGATCTCCTAGCCTGTGCCTCTGGGACTGGACAATTGCTTCAAGCATTCTTCAACCAGCAGATGCTGTTTA |
| *27* | *IFNG* | NM_000619.2 | 971-1070 | ATACTATCCAGTTACTGCCGGTTTGAAAATATGCCTGCAATCTGAGCCAGTGCTTTAATGGCATGTCAGACAGAACTTGAATGTGTCAGGTGACCCTGAT |
| 28 | *IFNGR1* | NM_000416.1 | 1141-1240 | CCCGGGCAGCCATCTGACTCCAATAGAGAGAGAGAGTTCTTCACCTTTAAGTAGTAACCAGTCTGAACCTGGCAGCATCGCTTTAAACTCGTATCACTCC |
| *29* | *IFNGR2* | NM_005534.3 | 800-899 | CAGTGGCCCTGAGCAATAGCACGAGGCCTGTTGTCTACCAAGTGCAGTTTAAATACACCGACAGTAAATGGTTCACGGCCGACATCATGTCCATAGGGGT |
| *30* | *IL1B* | NM_000576.2 | 841-940 | GGGACCAAAGGCGGCCAGGATATAACTGACTTCACCATGCAATTTGTGTCTTCCTAAAGAGAGCTGTACCCAGAGAGTCCTGTGCTGAATGTGGACTCAA |
| 31 | *IRF1* | NM_002198.2 | 16-115 | TTAGTCGAGGCAAGACGTGCGCCCGAGCCCCGCCGAACCGAGGCCACCCGGAGCCGTGCCCAGTCCACGCCGGCCGTGCCCGGCGGCCTTAAGAACCCGG |
| *32* | *IRF3* | NM_001571.5 | 1304-1403 | TCATGGCCCCAGGACCAGCCGTGGACCAAGAGGCTCGTGATGGTCAAGGTTGTGCCCACGTGCCTCAGGGCCTTGGTAGAAATGGCCCGGGTAGGGGGTG |
| *33* | *IRF5* | NM_002200.3 | 1846-1945 | GCCTGGCTCTCGGGAAATTCAGCCATGAGCAGGGAAAGAACTCTCCCAACCCTGGGGCCTAGCTGTATAGGAGGAATTGCCTAAGGGTGGCCCACTCTTG |
| 34 | *IRF7* | NM_001572.3 | 1764-1863 | CGCAGCGTGAGGGTGTGTCTTCCCTGGATAGCAGCAGCCTCAGCCTCTGCCTGTCCAGCGCCAACAGCCTCTATGACGACATCGAGTGCTTCCTTATGGA |
| *35* | *IRF9* | NM_006084.4 | 386-485 | GCACTCAACAAGAGTTCTGAATTTAAGGAGGTTCCTGAGAGGGGCCGCATGGATGTTGCTGAGCCCTACAAGGTGTATCAGTTGCTGCCACCAGGAATCG |
| *36* | *ISG15* | NM_005101.3 | 306-405 | CCCGGCAGCACGGTCCTGCTGGTGGTGGACAAATGCGACGAACCTCTGAGCATCCTGGTGAGGAATAACAAGGGCCGCAGCAGCACCTACGAGGTACGGC |
| 37 | *JAK1* | NM_002227.1 | 286-385 | GAGAACACCAAGCTCTGGTATGCTCCAAATCGCACCATCACCGTTGATGACAAGATGTCCCTCCGGCTCCACTACCGGATGAGGTTCTATTTCACCAATT |
| *38* | *JAK2* | NM_004972.3 | 1465-1564 | TTGATGTCAGTATTAAGCAAGCAAACCAAGAGGGTTCAAATGAAAGCCGAGTTGTAACTATCCATAAGCAAGATGGTAAAAATCTGGAAATTGAACTTAG |
| *39* | *C-JUN* | NM_002228.4 | 1543-1642 | CCTACGGCGCGGCCGGCCTGGCCTTTCCCGCGCAACCCCAGCAGCAGCAGCAGCCGCCGCACCACCTGCCCCAGCAGATGCCCGTGCAGCACCCGCGGCT |
| 40 | *JUNB* | NM_002229.2 | 1156-1255 | GCGCGCCTGGAGGACAAGGTGAAGACGCTCAAGGCCGAGAACGCGGGGCTGTCGAGTACCGCCGGCCTCCTCCGGGAGCAGGTGGCCCAGCTCAAACAGA |
| *41* | *JUP* | NM_002230.2 | 1076-1175 | CTCGTGCAGATCATGCGTAACTACAGTTATGAAAAGCTGCTCTGGACCACCAGTCGTGTGCTCAAGGTGCTATCCGTGTGTCCCAGCAATAAGCCTGCCA |
| *42* | *LYZ* | NM_000239.2 | 306-405 | ATGATGGCAAAACCCCAGGAGCAGTTAATGCCTGTCATTTATCCTGCAGTGCTTTGCTGCAAGATAACATCGCTGATGCTGTAGCTTGTGCAAAGAGGGT |
| 43 | *MNDA* | NM_002432.1 | 788-887 | CCAACGGCAGGTGGATGCAAGAAGAAATGTTCCCCAAAACGACCCAGTGACAGTGGTGGTACTGAAAGCAACAGCGCCATTTAAATACGAGTCCCCAGAA |
| *44* | *MX1* | NM_002462.2 | 1486-1585 | GCCTTTAATCAGGACATCACTGCTCTCATGCAAGGAGAGGAAACTGTAGGGGAGGAAGACATTCGGCTGTTTACCAGACTCCGACACGAGTTCCACAAAT |
| *45* | *NFKBIA* | NM_020529.1 | 946-1045 | GGATGAGGAGAGCTATGACACAGAGTCAGAGTTCACGGAGTTCACAGAGGACGAGCTGCCCTATGATGACTGTGTGTTTGGAGGCCAGCGTCTGACGTTA |
| 46 | *S100A8* | NM_002964.3 | 116-215 | CTGATAAAGGGGAATTTCCATGCCGTCTACAGGGATGACCTGAAGAAATTGCTAGAGACCGAGTGTCCTCAGTATATCAGGAAAAAGGGTGCAGACGTCT |
| *47* | *S100A9* | NM_002965.2 | 76-175 | AACATAGAGACCATCATCAACACCTTCCACCAATACTCTGTGAAGCTGGGGCACCCAGACACCCTGAACCAGGGGGAATTCAAAGAGCTGGTGCGAAAAG |
| *48* | *SAMD3* | NM_005902.4 | 1199-1298 | CATAATAACTTGGACCTGCAGCCAGTTACCTACTGCGAGCCGGCCTTCTGGTGCTCCATCTCCTACTACGAGCTGAACCAGCGCGTCGGGGAGACATTCC |
| 49 | *SOCS1* | NM_003745.2 | 1049-1148 | TAACTGTATCTGGAGCCAGGACCTGAACTCGCACCTCCTACCTCTTCATGTTTACATATACCCAGTATCTTTGCACAAACCAGGGGTTGGGGGAGGGTCT |
| *50* | *STAT1* | NM_007315.3 | 1796-1895 | TCCATCCTTTGGTACAACATGCTGGTGGCGGAACCCAGGAATCTGTCCTTCTTCCTGACTCCACCATGTGCACGATGGGCTCAGCTTTCAGAAGTGCTGA |
| *51* | *STAT2* | NM_005419.3 | 1391-1490 | GATTTGGGACTTTGGTTACCTGACTCTGGTGGAGCAACGTTCAGGTGGTTCAGGAAAGGGCAGCAATAAGGGGCCACTAGGTGTGACAGAGGAACTGCAC |
| 52 | *TLR4* | NM_138554.2 | 2571-2670 | ACTCAGAAAAGCCCTGCTGGATGGTAAATCATGGAATCCAGAAGGAACAGTGGGTACAGGATGCAATTGGCAGGAAGCAACATCTATCTGAAGAGGAAAA |
| *53* | *TLR8* | NM_016610.2 | 2311-2410 | TTTAACTGATAGCCTATCTGACTTTACATCTTCCCTTCGGACACTGCTGCTGAGTCATAACAGGATTTCCCACCTACCCTCTGGCTTTCTTTCTGAAGTC |
| *54* | *TNF* | NM_000594.2 | 1011-1110 | AGCAACAAGACCACCACTTCGAAACCTGGGATTCAGGAATGTGTGGCCTGCACAGTGAAGTGCTGGCAACCACTAAGAATTCAAACTGGGGCCTCCAGAA |
| 55 | *TNFAIP3* | NM_006290.2 | 261-360 | CAAAGCCCTCATCGACAGAAACATCCAGGCCACCCTGGAAAGCCAGAAGAAACTCAACTGGTGTCGAGAAGTCCGGAAGCTTGTGGCGCTGAAAACGAAC |
| *56* | *TYK2* | NM_003331.4 | 1981-2080 | GGCTGCTTGCTGAGGGCCGGGGATGACTGCTTCTCTCTGCGTCGCTGTTGCCTGCCCCAACCAGGAGAAACCTCCAATCTCATCATCATGCGGGGGGCTC |
| *57* | *ZBP1* | NM_001160419.2 | 681-780 | ATGAGGACAGCAAAAGATGTGAACCGAGACTTGTACAGGATGAAGAGCAGGCACCTTCTGGACATGGATGAGCAGTCCAAAGCATGGACGATTTACCGCC |

Supplementary Table 1. Custom NanoString nCounter® CodeSet probe panel.
