# Supplementary material for: Single-cell RNA sequencing of circulating immune cells supports inhibition of TNFAIP3 and NFKBIA translation as psoriatic arthritis biomarkers
Source: Front Immunol. 2025 Feb 7;16:1483393. doi: 10.3389/fimmu.2025.1483393 (PMC11842318; doi:10.3389/fimmu.2025.1483393)
Supplement: Supplementary file 11 [file Table2.docx]

| **Number of Cells** | **Samples** | **Pattern/TcRb** | **Fisher score** | **VDJdb TcRb** | **MHC A** | **EPITOPE GENE** | **EPITOPE SPECIES** |
| --- | --- | --- | --- | --- | --- | --- | --- |
| 4 | PSC2, PSA3, PSC2, PSC1 | PVR | 1.30E-05 | CASSQDPVRSNEKLFF | HLA-A*03 | IE1 | CMV |
| 2 | HC1, HC2 | LWAG | 1.80E-03 | N/A | N/A | N/A | N/A |
| 19 | HC2 | CASSDSGQETQYF | 1 | N/A | N/A | N/A | N/A |
| 10 | PSC2 | CASSLGFEQGGWTQYF | 1 | CASSLRGESSYNEQFF | HLA-B*07 | pp65 | CMV |
| 13 | PSA3 | CASSQRTEGETQYF | 1 | CASSQDLGLAGGETQYF | HLA-A*03 | IE1 | CMV |
| 39 | PSA1 | CASSLDGWDYNEQFF | 1 | N/A | N/A | N/A | N/A |
| 124 | PSA3 | CASSQDVWGGWSTGELFF | 1 | CASSQDGRRDVWTGELFF | HLA-A*03 | IE1 | CMV |
| 16 | PSA1 | CASAPSGSVDEQFF | 1 | N/A | N/A | N/A | N/A |
| 10 | PSA3 | CSAEGDRGRSETQYF | 1 | N/A | N/A | N/A | N/A |
| 13 | PSC3 | CASSLTGNRETQYF | 1 | CASSPLTSRETQYF | HLA-A*03 | IE1 | CMV |
| 31 | PSA1 | CASSLEGGTFYTEAFF | 1 | CASSLEGYTEAFF | HLA-A*02 | pp65 | CMV |
| 37 | HC2 | CASSPTMNTEAFF | 1 | N/A | N/A | N/A | N/A |

Supplementary Table 2. TCR clonotype clustering and predicted antigen specificity.
